# Low-Temperature In-Induced Holes Formation in Native-SiO_x_/Si(111) Substrates for Self-Catalyzed MBE Growth of GaAs Nanowires

**DOI:** 10.3390/ma13163449

**Published:** 2020-08-05

**Authors:** Rodion R. Reznik, Konstantin P. Kotlyar, Vladislav O. Gridchin, Evgeniy V. Ubyivovk, Vladimir V. Federov, Artem I. Khrebtov, Dmitrii S. Shevchuk, George E. Cirlin

**Affiliations:** 1 Alferov University, ul. Khlopina 8/3, 194021 St. Petersburg, Russia; gridchinvo@yandex.ru (V.O.G.); ubyivovk@gmail.com (E.V.U.); vfedorov.fl@mail.ioffe.ru (V.V.F.); dmitrii.shevchuk@gmail.com (D.S.S.); george.cirlin@mail.ru (G.E.C.); 2 Saint-Petersburg State University, Universitetskaya Emb. 13B, 198504 St. Petersburg, Russia; konstantin21kt@gmail.com; 3 ITMO University, Kronverkskiy pr. 49, 197101 St. Petersburg, Russia; khrebtovart@mail.ru; 4 Institute for Analytical Instrumentation RAS, Rizhsky 26, 190103 St. Petersburg, Russia; 5 Saint-Petersburg Electrotechnical University “LETI”, ul. Professora Popova 5, 197376 St. Petersburg, Russia

**Keywords:** nanowires, silicon, GaAs, self-catalyzed growth, molecular-beam epitaxy

## Abstract

The reduction of substrate temperature is important in view of the integration of III–V materials with a Si platform. Here, we show the way to significantly decrease substrate temperature by introducing a procedure to create nanoscale holes in the native-SiO_x_ layer on Si(111) substrate via In-induced drilling. Using the fabricated template, we successfully grew self-catalyzed GaAs nanowires by molecular-beam epitaxy. Energy-dispersive X-ray analysis reveals no indium atoms inside the nanowires. This unambiguously manifests that the procedure proposed can be used for the growth of ultra-pure GaAs nanowires.

## 1. Introduction

Nowadays III–V semiconductor nanowires (NWs) attract increasing attention due to recent advances made in their use as building elements for various electronic, optical, and biological applications [1,2,3,4]. Due to their ability to accumulate strain in two dimensions [5,6,7], NWs geometry is ideal for the monolithic integration of semiconductor materials with different lattice-mismatched substrates, which is important for achieving high-performance optoelectronic devices based on Si technology. A commonly used NWs fabrication technique relies on the Au-catalytic mechanism generally referred to as vapor-liquid-solid (VLS) growth [8,9]. However, Au is known to be a detrimental impurity in Si, limiting the integration of those nano-objects with a Si platform [10]. Moreover, the use of Au as a catalyst can lead to uncontrolled doping of III–V NWs [11,12]. On the other hand, successful attempts to grow self-catalyzed GaAs NWs on silicon [13,14,15] are very promising. However, GaAs NWs arrays synthesized on an unprepared Si surface are usually not homogeneous in terms of the height and diameter of NWs, which is critical for many applications. Plissard et al. [16] and Reznik et al. [17] have shown that the use of Si substrates with a SiO_x_ covering layer and electron-beam lithography technique for forming holes in SiO_x_ allows one to synthesize regular and homogeneous arrays of self-catalyzed GaAs NWs on a substrate surface. By changing the size and the distance between holes in SiO_x_, it is possible to control the morphological parameters of the NWs array, such as the density and the diameter of NWs. The synthesis of regular and homogeneous NWs arrays with controlled morphological properties is necessary for numerous applications. However, the high cost and time-consumption of the electron-beam lithography processes encourage researchers to find other ways to synthesize homogeneous and separated NWs. It was demonstrated in the work of I Morral et al. [18] that gallium adatoms interact with SiO_2_ forming sparse nanocraters on preexisting subnanometer pinholes. Küpers et al. [19] introduced a two-step growth method, where Ga droplets are pre-deposited on SiO_x_/Si substrates to form pinholes in SiO_x_ for subsequent NWs synthesis. In order to further improve the homogeneity and controllability of GaAs NWs density and diameters, Tauchnitz et al. [20] developed a three-step in-situ surface modification procedure for non-patterned native-SiO_x_/Si(111) substrates which decouples the Ga-induced hole formation in SiO_x_ from the following Ga-assisted growth of GaAs NWs. Koivusalo et al. [21] added two more steps to the technology described in [20]: crystallization of Ga droplets into GaAs by As_2_ exposure and spontaneous oxidation of the Si surface by air exposure outside the MBE setup. But in all these cases, substrate temperature reached 660–780 °C during the procedure before the initiation of NWs MBE growth. However, reduction of the maximum temperature of the NWs growth process is critical for the integration of III–V materials with silicon technology.

Here, we present a novel approach to low-temperature In-induced holes formation in SiO_x_ for GaAs NWs MBE growth. The temperature at which indium adatoms form holes in SiO_x_ and evaporate from the surface is significantly lower than for gallium ones. Moreover, indium droplets do not etch silicon and do not interact with it [22]. The above makes it possible to decrease the substrate preparation temperature to a lower level than typical GaAs NWs MBE growth temperature.

## 2. Materials and Methods

GaAs NWs were grown on non-patterned native-SiO_x_/Si(111) substrates using MBE setup Riber Compact 21 equipped with Ga, In effusion cells and a valved cracker source for the supply of As_4_. Firstly, the substrate was outgassed at 350 °C for 1 h in an ultrahigh vacuum and then transferred into the growth chamber with no vacuum brake. Afterwards, the substrate was heated to a temperature of 400 °C, and after temperature stabilization, the In source shutter was opened for 20 s to form In droplet on the substrate surface. The In flux was set at 0.4 ML/s according to previous calibrations. On the next step the substrate was kept at the same temperature for 15 min for the etching of the holes in SiO_x_ induced by indium droplets. In order to stop the etching of the holes, the In droplets were crystallized into InAs by 2 min As_4_ exposure. It was established previously that under similar growth conditions InAs nanoscale islands may be grown on Si(100) surface [23,24,25]. After that the substrate was annealed for 5 min at a temperature of 550 °C to evaporate the InAs completely from the substrate surface. In the following step aimed at the growth of the NWs the substrate temperature was increased to 600 °C for Ga deposition on the surface during 5 s with a 15 s pause to enhance Ga adatoms diffusion towards the holes and to form droplets inside them. Finally, GaAs NWs growth was performed under Ga-rich conditions by opening Ga and As shutters for 10 min. The gallium flux was constant throughout the experiment and was set at 1 ML/s according to the previous calibrations.

In order to evaluate the parameters of the holes that were formed in SiO_x_ layer, firstly, only In was deposited with following etching, crystallization, and evaporation. The surface morphology of the native-SiO_x_/Si substrate with holes after etching was studied using atomic force microscopy (AFM, BioScope Catalyst, Bruker, Santa Barbara, CA, USA). After NWs growth, the morphology of the grown NWs was studied by scanning electron microscopy (SEM, Supra 25, Carl Zeiss, Oberkochen, Germany). Microstructure and chemical composition of the grown NWs were investigated by transmission electron microscopy (TEM, Libra 200FE, Carl Zeiss, Oberkochen, Germany) with energy-dispersive X-ray (EDX) spectroscopy techniques (X-Max 80, Oxford Ins., High Wycombe, UK).

## 3. Results and Discussion

**Figure 1:**
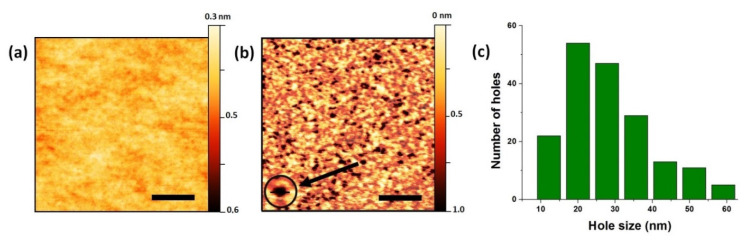
Typical AFM images of SiO_x_/Si(111) substrate surface: (**a**) initial and (**b**) after In droplets formation, etching, crystallization and evaporation of In from the surface. The scale bar corresponds to 250 nm. The insertion shows AFM image of a single hole in SiO_x_, which is approximately 35 nm in diameter (scale bar in the insertion corresponds to 50 nm); (**c**) Size distribution of holes in the SiO_x_ layer diagram.

At the preliminary stage of the growth, the holes in the SiO_x_ layer were unambiguously formed by In droplets etching. According to [26,27,28,29], a part of the deposited In adatoms on SiOx/Si substrate is oxidized because of oxygen migration from the SiOx overlayer. At this point, In oxides contribute to droplet self-organization by tuning In diffusion on the surface. During the following thermal annealing In oxidation plays the key role as it locally consumes the SiOx overlayer and drills nanoholes into SiOx until reaching the Si substrate. AFM profiles of the substrates surfaces (1 × 1 µm^2^) before and after etching, crystallization and evaporation of InAs from the surface are shown in Figure 1a,b. It is obvious that there are no holes in native-SiO_x_ which are clearly resolved after In pre-treatment. It is important to note that the holes formed are uniform in depth ~0.6 nm. Based on the measurement results, the average surface density of the holes amounts to 1.9 × 10^10^ cm^−2^. Figure 1c shows the distribution of holes in lateral size. The difference in the sizes of the holes can be related to the different sizes of In droplets formed on the substrate surface and their evolution during annealing. To conclude this stage, the described substrate preparation method allows one to form holes in native-SiO_x_ layers on Si(111) substrates.

**Figure 2:**
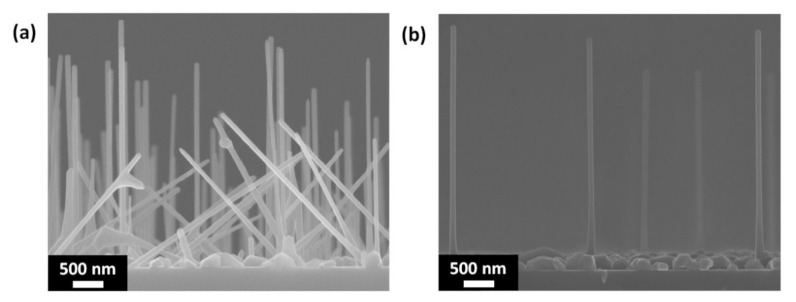
Typical SEM images of GaAs NWs grown on SiO_x_/Si(111) substrate: (**a**) without In pre-treatment; (**b**) with In pre-treatment.

Figure 2 shows typical SEM images of GaAs NWs grown on a native-SiO_x_/Si (111) substrate under the same growth conditions with and without In pre-treatment. As can be seen from the images, GaAs NWs without In pre-treatment follow different crystallographic directions and are inhomogeneous in morphological parameters. In turn, after In pre-treatment the NWs formed predominantly in the <111> direction. This means that NWs synthesis occurs epitaxially in the holes in SiO_x_/Si(111) surface. The average length of NWs is 3.5 μm, whereas the average surface density of NWs is about 2 × 10^−6^ cm^−2^. The difference in the densities of holes in SiO_x_ and grown NWs may indicate that the NWs growth was initiated not in all of the holes. Indeed, most of the NWs have a diameter bigger than 50 nm, which is typical for Ga-catalyzed NWs growth [13]. According to the AFM measurements, only a small fraction of the holes exhibits lateral sizes bigger than 50 nm, that is why the NWs surface density is much smaller than the surface density of the holes. It should be noted that the diameter of the NWs bottom is not absolutely homogeneous. As the length increases to 500 nm, the NWs diameter decreases from 85 nm to 55 nm and then remains constant up to the top of the NWs. It turned out that, similar to the MBE growth using etching by Ga droplets [19], other rather flat objects were formed on the substrate surface, in addition to GaAs NWs.

**Figure 3:**
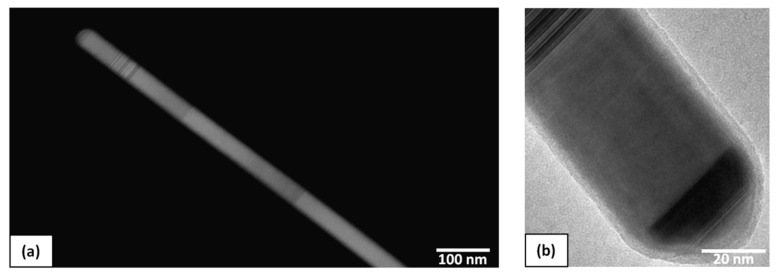
(**a**) HAADF-STEM image of a single GaAs NW; (**b**) HR TEM image of a single GaAs NW.

For studying structural properties of the NWs, they were transferred from the substrate onto the carbon mesh. As shown in Figure 3, the structure of the NWs is predominantly cubic, which is typical for self-catalyzed GaAs NWs growth [13].

**Figure 4:**
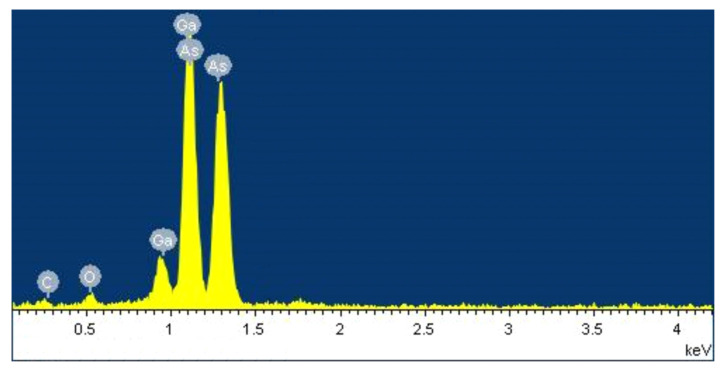
Typical EDX spectrum of GaAs NW measured at the center of the NW.

EDX analysis was carried out on several NWs. Figure 4 shows a typical EDX spectrum of GaAs NW. According to [30], peaks corresponding to In are observed in the EDX spectrum in the range from 3 to 4 keV. As can be seen from the figure, peaks corresponding to In are not observed in the EDX spectrum of grown NWs. Thus, indium was not detected along the entire length of NWs, which indicates its complete evaporation from the substrate surface before the GaAs NWs growth.

To conclude, we have shown the way to significantly decrease substrate temperature by introducing a procedure to create nanoscale holes in a native-SiOx layer on a Si(111) substrate via In-induced drilling. Using the fabricated template, we have successfully grown self-catalyzed GaAs nanowires by molecular-beam epitaxy. The fact that NWs formed in the <111> direction indicates that In droplets perfectly etch SiO_x_ down to the silicon surface, and hence the NWs growth occurs epitaxially directly in the holes. Energy-dispersive X-ray analysis reveals no indium atoms inside the NWs. This unambiguously manifests that the procedure proposed can be used for the growth of ultra-pure GaAs NWs.
